# Possible effects of an early diagnosis and treatment in patients with growth hormone deficiency: the state of art

**DOI:** 10.1186/s13052-017-0402-8

**Published:** 2017-09-16

**Authors:** Stefano Stagi, Perla Scalini, Giovanni Farello, Alberto Verrotti

**Affiliations:** 1 Health Sciences Department, University of Florence, Anna Meyer Children’s University Hospital, viale Pieraccini 24, Florence, Italy; 2 Department of Paediatrics, University of L’Aquila, L’Aquila, Italy

**Keywords:** Growth hormone, Growth hormone deficiency, Early treatment, Height, Adult height, Brain, Metabolism, Dwarfism, Early diagnosis, Hormone replacement therapy

## Abstract

Growth hormone deficiency (GHD) is a relatively uncommon and heterogeneous endocrine disorder presenting in childhood with short stature. However, during the neonatal period, the metabolic effects of GHD may to require prompt replacement therapy to avoid possible life-threatening complications. An increasing amount of data suggests the importance of an early diagnosis and treatment of GHD because of its auxological, metabolic, and neurodevelopmental features with respect to the patients diagnosed and treated later in life.

The available results show favourable auxological outcomes for patients with GHD diagnosed and treated with r-hGH early in life compared with those from patients with GHD who do not receive this early diagnosis and treatment. Because delayed referral for GHD diagnosis and treatment is still frequent, these results highlight the need for more attention in the diagnosis and treatment of GHD.

Despite these very encouraging data regarding metabolic and neurodevelopmental features, further studies are needed to better characterize these findings. Overall, the importance of early diagnosis and treatment of GHD needs to be addressed.

## Background

Growth hormone deficiency (GHD) is a relatively uncommon and heterogeneous disorder, in terms of aetiology, pathogenesis, age of diagnosis, and the cause of growth retardation and short stature [1]. The prevalence of GHD in childhood widely varies between 1/3480 and 1/30,000 children [2], even if the milder phenotype has a frequency of nearly 1:2000 [3].

In children, the main manifestation of GHD is represented by growth failure [1], and growth hormone (GH) ameliorates the short- and long-term height prognosis in these patients [4]. However, in children, GH may influence bone mineralization and bring about a large number of metabolic effects, involving lipid and glucose homeostasis and lean and fat mass [5].

The long-term growth response to GH treatment in GHD children may be conditioned by both pre-treatment and treatment-related factors, such as birth weight, baseline height standard deviation score (SDS), age at the onset of treatment, height at the start of puberty, treatment duration, target height (TH), mean frequency of injections, and doses of GH [6–8]. However, in a small subset of patients, GHD is recognizable in infancy or early childhood (i.e., < 3 years of age), and some data suggest that an early GHD diagnosis and a concomitant early start of treatment have proven effective in normalizing height [8–18].

It appears to be of great concern to consider early diagnosis and treatment as an advantageous and cost-effective strategy in achieving height improvement and a normal growth pattern during childhood compared with delayed start of treatment [19]. Additionally, the psychological benefits are greater, and the improvement in cost effectiveness is vast [9]. On the whole, published data suggest that the early diagnosis and treatment of GHD may have metabolic and neurodevelopmental consequences in childhood and adulthood [20].

In this review, we retrospectively evaluate data regarding early GHD diagnosis and treatment with r-hGH, examining their auxological, metabolic, and neurodevelopmental features with a focus on patients who have been diagnosed and treated later in life. For this study, relevant papers published in English were identified through systematic searches of the PubMed, EMBASE and Cochrane databases. Keywords in the literature search were entered in all combinations. Searches were augmented by manually reviewing the reference lists of all original articles and all systematic review articles, with each study being evaluated for inclusion.

## GH actions in the growth plate

The growth plate is a thin layer of cartilage localized between the epiphyseal and metaphyseal bone at the ends of the long bones. Longitudinal bone growth occurs at the growth plate by endochondral ossification, a process in which cartilage is first formed and then remoulded into bone tissue in a determined temporal and spatial organization. The growth plate consists of three principal layers: the germinal zone, proliferative zone, and hypertrophic zone [21]. At the epiphyseal end of the growth plate, the germinal zone contains the resting progenitors (stem cells), which differentiate into chondrocytes and progress through the proliferative zone. In this matrix-rich setting, the chondrocytes undergo cell divisions and arrange in columns that parallel the longitudinal axis of the bone. Immediately after proliferating, chondrocytes lose their capacity to divide and start to differentiate into prehypertrophic chondrocytes, increasing in size at same time [22]. They then further undergo terminal differentiation into hypertrophic chondrocytes, which have a round appearance and secrete large amounts of matrix proteins [23, 24]. The mineralization process, in combination with low oxygen tension, determines the changes in the environment that allow vascular invasion from the marrow of the metaphysis, thereby enabling the recruitment of osteoclasts and differentiating osteoblasts that remodel the newly formed cartilage into bone tissue [25, 26]. Subsequently, the mineralized chondrocytes undergo programmed apoptosis, leaving a platform for new bone formation. The final effect is that new bone tissue is progressively created at the bottom of the growth plate, resulting in bone elongation. During puberty and its associated rapid growth, the processes of cellular addition and replacement are coupled so that the width of the growth plate remains constant [27], while at the end of the growth period, the growth plate narrows and finally disappears as a result of systemic control and the fusion of the epiphysis with the metaphysis. However, it is now believed that regulation is intrinsic to the growth plate and that growth plate fusion may not precede, but rather follow, the cessation of growth [28, 29].

In children, maintenance of growth is a complex process that is regulated by a multitude of genetic, hormonal, environmental and nutritional factors [30, 31]. The major systemic hormones that regulate longitudinal bone growth during childhood are GH and insulin-like growth factor 1 (IGF-I), thyroid hormone (T_3_ and T_4_), glucocorticoids, and during puberty, sex steroids (androgens and oestrogens) [32, 33]. However, two of the most important regulators of postnatal bone growth are GH and IGF-I.

Growth hormone and IGF-I are potent stimulators of longitudinal bone growth [34]. Pituitary adenoma in childhood or adulthood causes excessive GH secretion, leading to gigantism or acromegaly, respectively [35, 36]. Conversely, GH deficiency or insensitivity due to GH-receptor mutations or defects in GH signalling pathways or in the formation of GH-secretory cells all result in severe dwarfism [37–42]. Data on patients with untreated isolated GH deficiency suggest that it leads to an average final height SDS of −4.7 (−6.1 to −3.9), [43].

In the early 1950s, Salmon and Daughaday described the somatomedin hypothesis. They proposed that the effects of GH on bone longitudinal growth were indirect and mediated by liver-derived IGF-I [44, 45], which activates chondrocyte proliferation in the growth plate. However, further studies have led researchers to reconsider this theory, since it was also demonstrated that GH can directly stimulate linear bone growth by local action on the growth plate. Isaksson et al. found that local GH injection into the tibial bone significantly stimulates longitudinal growth, whereas the contralateral tibia did not show this increase [46]. Studies in rats demonstrated pronounced tibial bone growth in the GH- or IGF-I-injected growth plate compared with the contralateral vehicle-injected growth plate [47, 48]. Furthermore, local injection of GH regulates the number of chondrocytes expressing IGF-I in rats, suggesting that part of the local action of GH on the growth plate may be mediated by increased local production of IGF-I [49]. Those observations led to the dual effector theory of GH/IGF-1 action at the growth plate, which states that GH acts directly on the germinal zone precursors of the growth plate to stimulate the differentiation of chondrocytes and then amplify local IGF-1 synthesis, which in turn induces the clonal expansion of chondrocyte columns in an autocrine/paracrine manner [50–53]. In support of the dual-effector theory but also suggesting a direct role of IGF-I on the growth plate, there are studies on mice with an inactivated GHR gene and studies on double knockout mice for GHR and IGF-I. The GHR/IGF-I double mutants were smaller than either the GHR or IGF-I single mutants, suggesting that both GH and IGF-I play a key role in the complex mechanism that leads to longitudinal growth [54].

## Metabolic actions of GH

The first reports on significant changes in body composition in child hypopituitary dwarfs were reported over 40 years ago [55, 56]. Afterwards, if we focus on the effect of GHD on body composition, several studies have confirmed its association with an increase in body fat and decreased lean body mass (LBM) in children [57–60]. Treatment with r-hGH results in an early normalization of body fat percentage within 6 months (58) and a steady increase in LBM during a 2- to 6-year treatment period [57, 60], while after a 6-month discontinuation of therapy, a rapid change in body composition can be already assessed in adult GHD, with an increased amount of fat mass and a decrease in LBM [61]. In particular, GHD adults display disproportionate increases in central abdominal fat. In a study conducted on adolescents with GHD at 6 and 12 months after discontinuation of r-hGH therapy, the authors registered no change in LBM [62]. These data appear to be in contrast with those on adults, but at the end of the study, although linear growth was virtually complete, full body compositional maturation had not occurred yet. It is therefore indicated that GH cessation was associated with a static LBM, while the group receiving GH had a continuing accrual of LMB. Using a CT scan, Rutherford et al. [63] demonstrated that when r-hGH was stopped for 12 months in adolescents treated for isolated GHD, body fat and muscle size increased, while integrity and strength decreased. These patients have increased levels of total and LDL cholesterol [64] and a higher prevalence of impaired glucose tolerance [65]. Additionally, they have been found to have a greater prevalence of atheromatous plaques in the carotid and femoral vessels [66] and a two-fold increase in cardiovascular mortality [67]. It is well known that GH has significant effects on fat, glucose and protein metabolism. A number of studies have shown that GH increases lipolysis and fat oxidation, reduces glucose oxidation and impairs insulin action [68–70]. GH lowers protein oxidation and stimulates protein synthesis. It is likely that the protein-sparing effect of GH, together with its effects on stimulating protein synthesis, contribute to the increase in LMB during treatment [71, 72]. However, in acromegaly, energy metabolism is characterized by a trend towards higher carbohydrate oxidation in the basal state and a greater rate of carbohydrate oxidation following oral glucose [73]. The findings of increased carbohydrate oxidation and decreased lipid oxidation are the opposite of those observed after short-term GH administration. IGF-I, but not glucose and/or insulin, was significantly related to basal and post-glucose carbohydrate oxidation. Studies with recombinant IGF-I in men have shown that this growth factor stimulates glucose disposal and increases carbohydrate oxidation [74]. Lewitt et al. have provided strong evidence in vivo that IGF-I and its related binding proteins play a significant role in glucose homeostasis [75].

## GH effects on neurodevelopment

In humans, growth hormone binding sites, indicative of GH receptor (GH-R) expression, are located on neurons, astrocytes, oligodendrocytes and microglia, and IGF-I was found to stimulate neuron growth, dendritic arborisation and synaptogenesis [76, 77]. In particular, GH-Rs were demonstrated in their highest concentration in the choroid plexus, thalamus, hypothalamus, pituitary, putamen and hippocampus [77], while IGF-I receptors are highly expressed in the hippocampus, amygdala, caudate nucleus, prefrontal cortex and parahippocampal cortex [76, 78]. There is also evidence that both GH and IGF-I are transported across the blood-brain barrier via transcytosis [79]. In addition, GH mRNA can be found in the central nervous system (CNS), suggesting that GH also acts in an autocrine/paracrine manner [80]. Interestingly, a reduction in GH binding sites within the brain has been observed with increasing age [77, 81]. Moreover, amongst elderly subjects, those with higher concentrations of IGF-I have been demonstrated to perform better on cognitive function tests and have lower rates of cognitive decline, suggesting that the GH-IGF-I axis affects cognitive performance throughout life [82].

The somatotropic axis plays a central role in the development and growth of the CNS. IGF-I intraventricular infusion in rats improves cognitive performance, and the inhibition of IGF-I binding to its receptors has been shown to lead to cognitive impairment, particularly in learning and reference memory [83]. Foetal cord IGF-I and IGF-binding protein-3 (IGFBP-3) concentrations have been related to head circumference at birth [84], and children with growth hormone receptor mutations have varying cognitive phenotypes [85, 86]. Similarly, serum IGF-I concentrations correlate positively with verbal intelligence in childhood [87].

In addition, there are several studies assessing a neuroprotective role of GH and IGF-I, either in combination or alone. For instance, after spinal cord injury, a decreased production of GH and IGF-I can be found [88], and in studies in rats with hypoxic brain damage, Sheepens et al. [89], found augmented immunoreactivity for GH in areas with strong cell loss. After intraventricular application of GH, the amount of neuronal loss was reduced in the frontoparietal cortex, hippocampus and dorsolateral thalamus but was left unchanged in the striatum, reflecting the regional distribution of GH receptors mentioned above, suggesting that these particular neuroprotective effects are mediated directly by GH alone.

Given these previous findings, it is unsurprising that several neuropsychological studies have documented impairments in cognitive functioning (memory and attention) in GHD. Despite a normal intelligence quotient (IQ), short children often show educational impairment, particularly in the domain of reading, spelling and arithmetic, and children with GHD show learning and attention deficits [90] and disturbances in visual-motor integration [91]. An impaired social status in patients with childhood-onset GHD has been reported compared with short and normal controls [92, 93], and women with untreated GHD exhibited lower scores on neuropsychological tests than healthy controls [94]. In a meta-analysis on adults with childhood- or adult-onset GHD, Falletti et al. demonstrated a link between GH and cognitive performance, which improved in GHD patients when growth hormone was replaced [95].

The effects of growth hormone substitution were also studied by Deijen et al., who evaluated the effects of three different doses of GH (1, 2, and 3 IU/m2/day) compared with a placebo treatment over six months, and then in an open-label phase over 18 months. In the first six months, they found improvements in memory function compared with placebo only in the groups with high GH doses, leading to supranormal IGF-I levels. In the next 18 months (open-label phase), they found improvements in memory functions at all doses [96]. This study confirmed the positive findings of GH substitution on cognitive abilities in previously conducted studies with small patient numbers [97, 98]. More recently, Webb EA et al. [99], evaluated the effect of GHD on brain structure and cognition in 15 children (mean age 8.8 years) compared to a group of children with idiopathic short stature. Compared with controls, children with isolated GHD had lower IQ, particularly in the area of verbal comprehension, which is correlated with IGF-I levels, and motor function, thus highlighting the link between GH and cognitive performance. Therefore, poor performance can be ameliorated with GH treatment.

## Early diagnosis and treatment of GHD

Although most children with clinical evidence of congenital GHD are diagnosed in the first months or years of life, some are recognized relatively late in childhood, at a time when short stature becomes clearly evident. In contrast, there is a subset of patients whose GHD is recognized before 3 years of age. In these patients, short-term and long-term studies have shown a marked early catch-up growth and a significant amelioration of height with an early start of r-hGH treatment [19]. However, data on the metabolic and neurodevelopmental differences in these two cohorts (early and late diagnosis of GHD and treatment) are very poor [20].

## Auxological outcomes of early GH treatment

In the past, reports have suggested that early diagnosis and treatment of GHD patients lead to a greater improvement in height gained, even if the final height remained near 2 SD below the mean population [100–106]. However, in 1995, Boersma et al. evaluated 26 children (7 with isolated GHD and 19 with GHD in combination with other pituitary deficiencies) who had started GH treatment before the age of 3 years and reported that early treatment of GHD may to lead to adequate catch-up growth, stressing that the early diagnosis and treatment before the statural impairment became evident was essential [14]. In the same paper, Boersma et al. reported how height SDS correlated positively with injection frequency and height SD score at start of treatment, noting that past treatment regimens, for example, 2 or 4 injections per week, could be responsible for failure to complete height gain [14]. In contrast, in children treated with 6 or 7 injections per week, the initial height at the start of treatment was reported to be crucial, since those with an initial height SDS between −2 and −4 showed a remarkable catch-up growth, whereas children with an initial height SDS < −4 did not reach full catch-up growth, although the study follow-up was only 4 years [14].

At the same time, Arrigo et al. studied 23 patients with early-onset GHD, 8 of whom had multiple hypophyseal deficiencies; these patients were treated before 5 years of age with GH and followed up for more than 3 years [15]. The data clearly showed that the height SDS at the end of the study was significantly better than in prior GH treatments, and the predicted height did not differ from the target height [15]. However, in the 6 (26%) patients showing a height < −2 SDS at the end of the study, only 2 showed a height > −4 SDS prior to GH treatment. Conversely, many patients showed a considerable height gain during the study (for example, in patient 1 and 4, the Δheight gain was 6.8 and 6.7 SDS, respectively) [15], not completely confirming the hypothesis of Boersma et al. [14]. It is interesting to note that the authors adjusted the GH doses periodically (every 3 months) during the first years of life [15]. These data were confirmed by another study, involving 23 patients with GHD treatment starting before the age of 5 and extending for at least 9 years until adulthood. These patients attained an adult mean height that did not differ from the midparental height (−0.9 vs. -0.7 SDS), because only three patients failed to achieve a final height within the target range [16]. It is meaningful to note that their height outcome correlated negatively with chronological age at the initiation of GH treatment and positively with height at puberty onset [16], stressing the importance of early diagnosis for both reduced height loss at the beginning of GH treatment and height prognosis at the onset of puberty, which should always be considered in negative terms in relation to stature recovery. However, other data come from 49 French patients with GHD [10], who started GH treatment before 1 year of age and were treated for a mean of 8.0 ± 3.6 years, reaching a mean height of −0.4 SDS with a Δheight gain of 3.11 ± 2.06 SDS (exceeding 4 SDS in 19 patients). The authors reported that the catch-up growth was maximal during the first 3 years and during puberty. Even if the patients showed an acceptable growth spurt, no further catch-up growth was reported for patients who reached or exceeded their target size [10].

Furthermore, in a large prospective long-term study evaluating 19 children with isolated GHD and 30 children with multiple pituitary hormone deficiency treated before the age of 3 years with daily subcutaneous injections for 3–5 years, the authors demonstrated that 84% of the treated patients reached a height above 2 SDS below the mean after 5 years of treatment [17]. These patients were divided into two groups according to their height SDS for chronological height at the start of GH treatment: group A consisted of patients with an initial height within the −2 SDS, whereas group B consisted of patients with initial growth retardation (> −2 SDS) [17]. Both group A (the mean height significantly improved by −2.1 ± 0.6 at the start of GH treatment to 0.5 ± 0.8 after 5 years) and B (from - 3.6 ± 1.0 at the start of the GH treatment to 0.9 ± 1.2 after 5 years) showed a significant change in annual mean height SDS for chronological age during treatment until the fourth year of treatment (the fifth year was not evaluated due to the small number of patients) [17]. However, the evaluation of variables that could predict the total height gain after 5 years of treatment showed that only the height SDS at the start of therapy was significant, indicating that most infants with growth retardation had better catch-up growth [17]. These data strongly supported the need to start early GH treatment in GHD, before growth retardation becomes evident [17].

These results were also confirmed by other studies involving patients treated before the age of 2 [8, 18]. In particular, the authors discussed pre-treatment factors possibly affecting the catch-up growth during GH treatment in GHD children, such as mid-parental height and severity of GHD [107], pre-treatment height and bone age (BA) delay at treatment onset [108]. Interestingly, in a homogeneous population, Wasniewska et al. reported that height gain during this 7-year lasting study appears to be related to the difference between target height (TH) and pre-treatment height and BW [8].

Interestingly, Ranke et al. evaluated 265 children with idiopathic GHD treated before the age of 3, compared with 509 children treated after 7 years of age, and showed that after the first year of GH treatment, the height gain was significantly higher per GH dose unit in very young than in older children. The author suggested that the early detection and treatment of GHD is a cost-effective strategy and results in greater improvements in height and growth velocity compared to a delayed treatment start in childhood [9].

Finally, in another recent study involving 47 GHD patients, in whom the administration of r-hGH was started at or before the age of 2 and consequently followed up to near-adult heights (NAH), the authors reported that treatment was very effective, since at the beginning of the study, and the height was −2.3 SDS, whereas after 5 and 10 years of age and at NAH, the stature reached −0.6, −0.3, and −0.4 SDS, respectively, which was comparable to the TH [12]. Importantly, the authors showed that a normal pattern of linear growth was achieved during childhood (before the age of 10) and that no additional gain in NAH SDS was realized during puberty, consistent with the statement that sensitivity to GH is greater during childhood and that therapeutic efforts to maximize height should be concentrated in the prepubertal years [12].

## Metabolic outcomes

Despite the absence of overt abnormal findings at baseline, it is suggested that GHD children, even at a young age, may show subtle metabolic changes that may adversely affect their future metabolic and atherogenic profile.

In these respects, the available data are mainly derived from findings in patients with Prader-Willi syndrome (PWS), a genetic condition frequently presenting hypothalamic dysfunctions responsible for GH and thyroid-stimulating hormone (TSH) deficiencies, central adrenal insufficiency and hypogonadism [109]. It has been demonstrated that the PWS patients receiving early r-hGH treatment (begun prior to the age of 2) show improvements in body composition, motor function, height, and lipid profiles compared to those who were untreated [110]. However, in PWS, early r-hGH treatment seems to be unrelated to a significant increase in fasting insulin and HOMA-IR [111].

These results seem to be confirmed in subjects with GHD and optic nerve hypoplasia who had enrolled in studies around 30 months of age and had undergone early treatment with r-hGH. These subjects showed a reduction in body fat percentage and an improvement in lipid levels compared to untreated controls [112]. However, longitudinal studies involving more cases, especially patients with isolated GHD, are needed.

Interestingly, data derived by studies conducted in GHD dwarf rats have suggested that untreated early-onset GHD may to lead to significant impairment of left ventricular (LV) diastolic function and reductions in cardiac size by adulthood. In this model, early GH substitution increased and sustained the cardiac concentration of sarco/endoplasmic reticulum Ca^2+^-ATPase (SERCA2) and preserved cardiac morphology, limiting the onset of overt diastolic dysfunction [113].

However, it remains to be established whether the previously discussed issues may place patients at higher risk for cardiovascular disease later in life or reverse the long-term effects of r-hGH treatment.

## Psychological and neurodevelopmental outcomes

Recent studies have conclusively shown that GH and IGF-1 receptors are located throughout the brain, particularly in regions related to learning and memory, such as the hippocampus [99]. In addition, a large amount of data suggest that GHD and reduced IGF-1 levels may correlate with decreased cognitive ability [99], as shown in animals with GHD presenting with smaller brain volumes with reduced neuronal and glial proliferation [99].

In studies involving animal models with congenital GHD, such as Snell (*Pou1f1* deficient) [114], and *little (Ghrhr*-deficient) mice [115], the authors described the presence of hypomyelination and poor neuronal growth and synaptogenesis. These features appeared to be restored with postnatal GH administration [114; 115]. However, such findings were not confirmed by others [116] or suggested to be related to compensatory upregulated production of local IGF [117].

In humans, GH-treated infants and toddlers seem to be more alert and energetic, as reported by their families, and an increased rate of language and cognitive development was reported in GH-treated patients, even if such results may only be due to increased muscle tone [118]. However, some data are consistent with the finding that children with isolated GHD have improved intellectual development if they are treated before the age of 5 years [119] and present with improved attention, anxiety, social competence, and thought impairment after 3 years of treatment, even if this aspect was more pronounced for isolated GHD patients than for those with idiopathic short stature [120].

Interestingly, in a recent study evaluating the cognitive functioning in 15 prepubertal PWS patients aged 3.5 to 14 years, the authors reported that GH treatment can prevent the deterioration of some cognitive skills in the short term and significantly improve abstract reasoning and visuospatial skills during long-term r-hGH treatment. Furthermore, children with a greater deficit can benefit more from GH treatment [121]. This finding is in line with those of animal studies in which *dwarf* rats treated early with GH, therefore rendering them GH-replete in puberty but deficient as adults, displayed a spatial learning performance comparable to that of heterozygous animals, whereas a longer supplementation did not confer additional benefits, suggesting a “window period” to be able to obtain such benefits [122].

Regarding the impact of childhood GHD on cognition, published data outline how GHD children with low peak GH to stimulation or low GH levels in nocturnal GH secretion may present reduced performance in a visual motor psychological test [123]. Of note, in another study involving 79 GHD children evaluated after 1 and 3 years of r-hGH treatment, IQ scores at baseline were within the normal range, even if GHD children older than 7 years of age at first assessment had a mean performance IQ slightly below average [124].

Therefore, given the demonstrated effects of GHD on brain structure, cognition and possibly behaviour, an early diagnosis of GHD is a critical component of the work-up of these endocrine disorders.

## Conclusions

In conclusion, the available auxological results show a favourable auxological outcome, comparable to the TH, for patients with early-diagnosed GHD treated with r-hGH. GH retains good efficacy in very young patients, and early-diagnosed patients with GHD have a responsiveness to r-hGH that is much greater than that of older patients. Furthermore, early detection and treatment of GHD is a cost-effective strategy. Since delayed referrals for GH diagnosis and treatment still occur frequently, careful evaluation is needed when GHD is suspected, given the importance of early diagnosis and treatment.

Moreover, these preliminary but very interesting data about early GHD diagnosis and treatment and their possible effects on metabolism require confirmation in wider studies. Therefore, data highlighting the action of GH and IGF-1 deficiency on the brain structure, psychological and neurodevelopmental features of GHD patients, as well as the possible effects of GH treatment, seem to assume a “time” dependence of GHD diagnosis, thus stressing the importance of earlier diagnosis and treatment.
